# Maternal and Antenatal Complications in Pregnancies Complicated by Fetal Intra-Abdominal Umbilical Vein Varix

**DOI:** 10.3390/jcm15197444

**Published:** 2026-09-24

**Authors:** Kathy Zhang-Rutledge, Anushka Chelliah, Nikolaos Zacharias, Monique Sutherland, Kirsten Emanuel, Olaide Ashimi Balogun

**Affiliations:** Obstetrix Maternal-Fetal Medicine Specialists of Houston, Houston, TX 77054, USA; anushka.chelliah@pediatrix.com (A.C.); nikolaos.zacharias@pediatrix.com (N.Z.); monique.sutherland@pediatrix.com (M.S.); kirsten.emanuel@pediatrix.com (K.E.); olaide.ashimibalogun@pediatrix.com (O.A.B.)

**Keywords:** fetal intra-abdominal umbilical vein varix, fetal growth restriction, fetal weight, oligohydramnios, polyhydramnios

## Abstract

**Background/Objectives**: Prior research suggests that fetal intra-abdominal umbilical vein varix (FIUVV) is associated with an increased risk of structural anomalies, aneuploidy, and poor obstetrical outcomes such as growth restriction and demise. Data in prior studies, however, are limited. The aim of this study is to determine the natural progression of FIUVV and its association with maternal and antenatal complications. **Methods**: This is a retrospective cohort study of singleton pregnancies that received an ultrasound between July 2020 and June 2023. FIUVV was diagnosed based on the presence of a focal intra-abdominal vein dilation measuring >9 mm or >50% wider than the diameter of the intra-hepatic umbilical vein. Controls were matched 1:1 based on same date as the cases and by similar gestational age. Maternal and fetal outcomes were collected. **Results**: A total of 105 pregnancies complicated by FIUVV met inclusion criteria, and 105 controls were identified. Diagnosis was most commonly obtained at 32 gestational weeks with average dilation at 11.2 mm. Concurrent structural defects were detected in 19 (18.1%) fetuses. Compared to controls, pregnancies complicated by FIUVV had similar rates of diabetes, hypertensive disorders, COVID-19, active smoking, and abnormal genetic screening. There were also similar rates of fetal growth restriction, large-for-gestational-age fetuses, and oligohydramnios. Pregnancies complicated by FIUVV were more likely to have polyhydramnios compared to controls (*p* < 0.01). **Conclusions**: In the large cohort of FIUVV, outcomes were favorable. FIUVV was not associated with common maternal co-morbidities. Polyhydramnios was more common in pregnancy complicated by FIUVV, but fetal growth abnormalities were similar compared to controls.

## 1. Introduction

Fetal intra-abdominal umbilical vein varix (FIUVV), a focal dilation in the umbilical vein, is an uncommon prenatal ultrasound finding [1]. Throughout gestation, the diameter of the intra-abdominal portion of the umbilical vein increases linearly from 2 mm at 15 weeks of gestation to 8 mm at term [2]. The intra-abdominal portion of the umbilical vein is considered dilated if the diameter is >9 mm or >50% wider than the intra-hepatic portion of the umbilical vein. On prenatal ultrasound, FIUVV appears as an anechoic, cyst-like structure seen in the upper abdomen and typically involves the intra-abdominal and extra-hepatic portion of the umbilical vein [2]. Color Doppler can aid the diagnosis of FIUVV by revealing linear venous flow and continuity with the umbilical vein, findings that can help differentiate from normal structures such as the gallbladder or stomach and other pathological structures such as urachal cysts or mesenteric cysts. FIUVV may be an early manifestation of elevated venous pressure on a region of the umbilical vein wall that is intrinsically weaker due to a lack of supporting surrounding structures.

Early studies have found that FIUVV is associated with other concurrent structural anomalies, aneuploidy, and poor obstetrical outcomes such as fetal growth restriction and intrauterine demise [2,3,4,5,6,7,8]. Isolated FIUVV seems to have, in general, favorable outcomes; however, fetal growth restriction remains a risk [9,10,11,12].

The majority of studies on FIUVV are retrospective and observational. As a result, recommendations for the management of pregnancy with FIUVV are based on low levels of evidence. Following the available literature, our institution performs a detailed anatomy survey, if not already completed, serial growth ultrasounds, and additional weekly antenatal surveillance in the third trimester in pregnancies with FIUVV. Whether this close surveillance impacts fetal outcomes remains unknown.

The purpose of this study is to characterize the natural progression of FIUVV and to assess for an association between FIUVV and obstetrical complications.

## 2. Materials and Methods

This study was performed at Obstetrix Maternal-Fetal Medicine Specialists clinic, a referral-based maternal-fetal medicine practice, in Houston, Texas. The Institutional Review Board at Pediatrix Medical Group and the University of Houston School of Medicine approved the study. It examined a retrospective cohort of women with singleton gestations who underwent an ultrasound scan between July 2020 and June 2023 with pregnancies complicated by FIUVV. FIUVV was diagnosed based on the presence of a focal intra-abdominal vein dilation measuring >9 mm or >50% wider than the diameter of the intra-hepatic umbilical vein. To maximize the sample size, we included all eligible patients during the study period rather than performing an a priori sample size calculation. The control cohort comprised singleton pregnancies without suspected FIUVV matched 1:1 by ultrasound exam date and gestational age of the pregnancy within 2 weeks. If more than one control patient met the matching criteria, then the control patient with the closest gestational age to the study patient was selected.

We collected maternal demographic data including age, pre-pregnancy or first trimester body mass index, race/ethnicity, parity, medical comorbidities (i.e., diabetes, hypertension, COVID-19, smoking status), and gestational age at FIUVV diagnosis. The fetal outcomes collected were diagnosis of estimated fetal weight (EFW), presence and type of other structural anomalies, and genetic screening results (abnormal non-invasive prenatal screening or Quad screen). The estimated fetal weight (EFW) was determined using the Hadlock formula: head circumference, biparietal diameter, abdominal circumference (AC), and femur length [13]. EFW percentile was calculated using the Hadlock curve [14]. Fetal growth restriction (FGR) was defined as EFW or AC < 10th percentile, and large for gestational age (LGA) was defined as EFW > 90th percentile.

Pearson’s chi-square tests, paired *t*-tests, and Fisher’s exact tests, when appropriate, were applied to evaluate differences in maternal and neonatal outcomes, and the statistical results were reported as the relative risk with a 95% confidence interval. *p* < 0.05 was considered significant. Data was analyzed using STATA 16 (SPSS IBM, New York, NY, USA) 23.0 statistical software.

## 3. Results

During the study period, 26,356 unique gravid patients were scanned. A total of 105 singleton pregnancies were identified to have fetal intra-abdominal umbilical vein varix, and 105 singleton pregnancies were identified as controls.

With regard to maternal demographics, there were no differences between the case and control groups in maternal age and parity. The case group consisted of more patients who identified as Hispanic, White, or Black, and fewer patients who identified as Asian compared to the control group (Table 1). The rates of maternal co-morbidities (diabetes, hypertension, active smoking, COVID-19 infection) were also not statistically different between the two groups. Fifty-four (51.4%) of the cases were pregnancies with male fetuses, while 51 (48.6%) of the controls were pregnancies with male fetuses (*p* = 0.68). There were no differences in the rates of abnormal non-invasive prenatal testing (NIPT) between the case (2.8%) and control (2.9%) groups (*p* = 1.00).

Diagnosis of FIUVV was most commonly obtained at 32 gestational weeks, with average dilation at 11.2 mm (Table 2). Twenty-four (23%) of the cases of FIUVV “resolved” on follow-up ultrasound, while forty-three (41%) of the cases had further dilation of the FIUVV on subsequent scans. The mean largest FIUVV diameter was 12 mm and occurred at an average gestational age of 34 weeks. Earlier diagnosis (prior to 32 gestational weeks) was associated with further varix dilation (*p* < 0.01). The greatest amount of dilation was 12.2 mm when FIUVV was diagnosed before 32 gestational weeks and 12.0 mm when FIUVV was diagnosed after 32 gestational weeks. Nine (32.1%) of the twenty-eight fetuses diagnosed with FIUVV prior to 32 gestational weeks had other structural anomalies, while 10 out of 77 (13.0%) of fetuses diagnosed with FIUVV after 32 gestational weeks had other structural anomalies (*p* = 0.02).

Nineteen (18.1%) fetuses with FIUVV had concurrent structural anomalies (Table 3). The rate of associated anomalies was not statistically different between the FIUVV cohort and control group. The most common structural anomalies in the FIUVV cohort were urinary tract dilation and ventricular septal defect. When compared to isolated cases, fetuses with other structural defects had similar rates of FGR, LGA, oligohydramnios, and polyhydramnios. Two (1.9%) fetuses had abnormal genetic screening (NIPT) results, with the parents declining invasive prenatal genetic testing. One case was identified as Trisomy 21, and this fetus also had a thick nuchal fold and absent nasal bone. The other case was high risk for XXY syndrome or Klinefelter syndrome on NIPT, and this fetus did not have any structural anomalies on prenatal imaging. No cases of varix thrombosis or intrauterine demise were observed among this cohort of pregnancies complicated by FIUVV.

Compared to controls, pregnancies complicated by FIUVV had similar rates of fetal growth restriction, large-for-gestational-age fetuses, and oligohydramnios (Table 4). Eleven (10.5%) fetuses with FIUVV were diagnosed with FGR, while twenty-eight (26.7%) were LGA. FGR resolved in 2 (15.4%) of those 11 fetuses. LGA resolved in 4 (14.3%) of the 28 fetuses. Two (1.9%) fetuses with FIUVV had oligohydramnios, while twenty (19%) had polyhydramnios. Fetuses with FIUVV were three times more likely to have polyhydramnios compared to controls (*p* < 0.01).

## 4. Discussion

Our study reports the natural progression in a large contemporary cohort of pregnancies complicated by FIUVV and compares outcomes with a control population. We found the incidence of FIUVV to be 0.4% during our study period. Similar to prior reports, we found that the mean gestational age for diagnosis of FIUVV was in the early third trimester and FIUVV was associated with other structural anomalies. We did not show an increased risk for fetal growth disorders compared to controls. Interestingly, in our study, pregnancies with FIUVV had three times the rate of polyhydramnios compared to controls.

Early case studies report higher rates of poor fetal outcomes in fetuses with FIUVV. In 1992, Mahony reported a cohort of nine cases of FIUVV in which four fetuses subsequently died in utero and one fetus developed hydrops 2 weeks after FIUVV was detected [2]. Early systematic review reported that, among fetuses with FIUVV, 12% had chromosomal abnormalities, 5% developed hydrops, and 25% resulted in intra-uterine fetal demise (IUFD) [3]. Advancements in prenatal ultrasonography technology and adoption of ultrasound protocols have likely increased FIUVV’s detection. More contemporary studies show more reassuring but varied outcomes. Byers et al. found that FIUVV was associated with other anomalies in 28.8% of cases and aneuploidy (specifically Trisomy 21) in 5.8% of cases, and the authors suggested that FIUVV may be a marker for aneuploidy [4]. Further case reports reported the rate of associated structural anomalies and/or chromosomal abnormalities to range from 15 to 40% [5,6,10,15]. A recent meta-analysis covering the largest number of cases to date reported fetal anomalies in 15% of fetuses with FIUVV, with the most common anomalies involving the genitourinary tract [16]. Similarly, our study population showed that 17% of fetuses with FIUVV had other structural anomalies. Genitourinary tract anomalies are most common. In our study, 32% of fetuses diagnosed with FIUVV prior to 32 gestational weeks had structural anomalies, while 15% of fetuses with FIUVV diagnosed later (after 32 gestational weeks) had anomalies. This difference was not statistically different but may be clinically relevant. In our study, one fetus with FIUVV had suspected Trisomy 21, based on high-risk non-invasive prenatal testing (NIPT) and soft markers on ultrasound suggestive of Trisomy 21. Another fetus with FIUVV had NIPT results that were suggestive of Klinefelter syndrome. This fetus did not have any detectable structural anomalies on ultrasound, which is not expected in fetuses with Klinefelter syndrome. This patient declined invasive testing such as amniocentesis, and thus we do not have confirmatory genetic results for this fetus.

Previously, fetuses with FIUVV were also thought to be at increased risk for IUFD based on case reports and small cohort studies [5,17,18]. Larger cohort studies do not support this [15], and the reported cases of IUFD were typically in fetuses with other structural anomalies [4,19]. A proposed mechanism for IUFD in the FIUVV setting is the formation of thrombosis at the varix, which can obstruct venous return or increase cardiac pre-load, leading to heart failure [5,8]. While varix thrombosis has been reported in the literature, the incidence appears low [5,8,20,21]. Reassuringly, we did not observe any cases of varix thrombosis, intrauterine fetal demise, or hydrops in our study population.

Prior studies suggest FIUVV may be associated with an increased risk for fetal growth restriction or small-for-gestational-age birth weight [8,15,22]. Ghahremani reported the rate of fetal growth restriction to be 6.4% [16]. The rate of fetal growth restriction in our study population was 11%; however, this rate was not different compared to the controls. The rates of large for gestational age between FIUVV fetuses and controls were similar. Notably, we found a high rate of polyhydramnios in fetuses with FIUVV. Amniotic fluid level outcomes have not been collected in prior studies. Notably, we observed a significantly higher rate of polyhydramnios among pregnancies complicated by FIUVV compared with matched controls. To our knowledge, amniotic fluid abnormalities have not been systematically reported in previous studies of FIUVV, making this a novel finding. Although the mechanism underlying this association remains uncertain, several hypotheses may be considered. Both FIUVV and polyhydramnios may reflect altered fetal hemodynamics or underlying changes in fetal fluid homeostasis rather than a direct causal relationship. Venous dilation within the umbilical circulation may represent localized alterations in venous pressure or vascular compliance that could coexist with conditions affecting fetal fluid balance. Alternatively, the association may be mediated by concurrent fetal abnormalities that predispose to both venous dilation and abnormal amniotic fluid volume, although our subgroup analysis did not demonstrate a higher rate of polyhydramnios among fetuses with associated structural anomalies compared with those with isolated FIUVV. These findings suggest that the observed association is unlikely to be explained solely by the presence of congenital anomalies. Additional studies are needed to determine whether polyhydramnios represents a true component of the FIUVV phenotype or simply reflects a coincidental but unrelated antenatal finding.

Our study has several strengths. All ultrasounds were performed by maternal fetal medicine sonographers and interpreted by maternal fetal medicine specialists. While our data was collected from a single practice, we have multiple locations which serve a diverse patient population of varying age, races, and parity, which decreases sampling bias. Furthermore, the maternal demographics between the study and control groups were largely similar. One limitation of this study is its retrospective design. Since FIUVV is a relatively uncommon finding, achieving a substantial study size would be difficult in a prospective study. The size of the study population limits statistical power. A larger sample size may show statistical differences in rare outcomes such as varix thrombosis or intrauterine demise. Another limitation of this study is the lack of birth data and long-term follow up. Since our practice is consultative based and patients deliver at numerous hospitals in the community, it was not feasible to collect delivery outcomes and neonatal data. The rate of structural anomalies in the control group was about 10%, which is higher than the expected 3% rate of fetal anomalies in the general population [23]. The rate of fetal growth restriction in the control group was about 14%. This is most likely explained by the nature of our practice, as often patients are referred to our clinic due to suspected anomalies or suspected fetal growth disorder.

Our study on FIUVV showed that the incidence of associated structural anomalies is approximately 18%, which was not statistically different compared to a rate of 10% in the control group. Antenatal outcomes were generally favorable, with similar rates of fetal growth disorder, but FIUVV was associated with higher rates of polyhydramnios, which may reflect underlying fetal hemodynamic alterations.

It is important to have a standard management protocol after diagnosis of FIUVV. Previous publications recommend a targeted anatomy survey to evaluate for structural anomalies and markers for aneuploidy [11,16]. We concur with this recommendation, as the rate of structural and genetic abnormalities in our study population was approximately 15%. A fetal echocardiogram should be considered, especially if the cardiac views are not satisfactory on the anatomy survey, as cardiac anomalies constituted the second most common group of structural anomalies in our study. Genetic screening for aneuploidy should be offered, if not already done so, in cases of isolated FIUVV, and confirmatory genetic testing such as amniocentesis should be discussed and offered if there are concurrent structural anomalies. Serial scans after the diagnosis to monitor interval growth and amniotic fluid level is warranted since prior studies have shown an association with fetal growth restriction, and our study shows increased rates of polyhydramnios. The frequency of ultrasounds and necessity of antenatal testing remain unclear. Larger, contemporary studies, including ours, do not demonstrate an increased risk for IUFD; however, IUFD is a rare event. There is no evidence showing antenatal weekly testing such as weekly biophysical profiles or nonstress tests improves outcomes in FIUVV, as the etiology for IUFD is unlikely due to placental insufficiency. Nonetheless, serial ultrasound assessment every 1–2 weeks to assess amniotic fluid level and to visualize the FIUVV in gray scale and color Doppler may help “catch” worsening changes in the clinical course. The concurrent presence of FGR or amniotic fluid abnormalities prompts further work up and close follow up. Early authors have recommended delivery as early as 34–37 weeks for FIUVV [16,21]. Weighing the immediate and potential long-term risks of prematurity with reassuring contemporary data, there is likely little benefit to delivering earlier than 39 weeks of gestation if there are no other obstetrical indications. Long-term outcomes for fetuses with FIUVV are lacking and limited to only one small study, which reported a possible association between FIUVV and childhood developmental delays [24]. More studies exploring long-term physical and neurocognitive developmental outcomes are needed to guide prenatal counseling.

## Figures and Tables

**Table 1:** Comparison of maternal demographics, co-morbidities, and pregnancy characteristics.

	Case	Control	*p*-Value
Age (year) *	30 (0.6)	31 (0.6)	0.12
**Nulliparity**	35/105 (30.5%)	30/105 (36.2%)	0.38
BMI *	33 (0.6)	33 (0.7)	0.56
**Ethnicity**			**<0.01**
**Hispanic**	49/105 (47%)	39/105 (37%)	
**Race**			**0.03**
**Caucasian**	60/105 (57%)	56/105 (53%)	
**Black**	11/105 (11%)	8/105 (8%)	
**Asian**	3/105 (3%)	8/105 (8%)	
**COVID-19**	5/105 (4.8%)	3/105 (2.9%)	0.50
**Active smoking**	0	0	1.00
**Diabetes**	24/105 (22.9%)	19/105 (18.1%)	0.21
**Pregestational Diabetes**	3/105 (2.9%)	6/105 (5.7%)	
**Gestational Diabetes**	21/105 (20%)	13/105 (12.4%)	
**Hypertensive Disorder**	6/105 (5.7%)	11/105 (10.5%)	0.27
**Chronic Hypertension**	6/105 (5.7%)	8/105 (7.6%)	
**Gestational Hypertension or Preeclampsia**	0/105 (0%)	3/105 (2.9%)	
**Male Fetus**	54/105 (51.4%)	51/105 (48.6%)	0.68
**Abnormal NIPT**	2/72 (2.8%)	2/68 (2.9%)	1.00

* Data presented in mean (standard deviation) NIPT, non-invasive prenatal testing.

**Table 2:** Natural clinical course in FIUVV.

**GA at diagnosis (weeks)**	32.9 (0.3)
**EFW at diagnosis (grams)**	2211 (77)
**EFW % at diagnosis**	56 (3)
**FIUVV diameter at diagnosis (mm)**	11.2 (0.2)
**Largest FIUVV diameter (mm)**	12.0 (0.2)
**GA at largest diameter (weeks)**	34.0 (0.3)

Data presented in mean (standard deviation) GA, gestational age; EFW, estimated fetal weight; FIUVV, fetal intra-uterine umbilical vein varix.

**Table 3:** Characteristics of associated structural anomalies.

**Central nervous system**	Mega cisterna magna, Mega cisterna magna and ventriculomegaly
**Cardiovascular**	Absent inferior vena cava and Foramen ovale aneurysm, Ductal arch aneusym, Hypertrophic cardiomyopathy, Tetrology of Fallot, Aortic coarctation, Ventricular septal defect (3)
**Gastrointestinal**	Echogenic bowel
**Genito-urinary**	Urinary tract dilation (6)
**Umbilical cord**	Single umbilical artery

**Table 4:** Comparison of antenatal outcomes.

	Case	Control	RR	95% CI	*p*-Value
**Associated Anomalies**	19/105 (18.1%)	11/105 (10.5%)	1.73	0.87–3.45	0.12
**Fetal Growth Restriction**	11/105 (10.5%)	15/105 (14.3%)	0.73	0.35–1.52	0.40
**Large for Gestational Age**	28/105 (26.7%)	27/105 (25.7%)	1.04	0.66–1.63	0.88
**Oligohydramnios**	2/105 (1.9%)	4/105 (3.8%)	0.50	0.09–2.67	0.68
**Polyhydramnios**	20/105 (19%)	6/105 (5.7%)	**3.33**	**1.39–7.97**	**<0.01**

## Data Availability

The data presented in this study are available on request from the corresponding author.

## Abbreviations

The following abbreviations are used in this manuscript.

FIUVV	Fetal Intraabdominal Umbilical Vein Varix
COVID-19	Coronavirus Disease 2019
EFW	Estimated Fetal Weight
AC	Abdominal Circumference
NIPT	Non-invasive Prenatal Testing
FGR	Fetal Growth Restriction
LGA	Large for Gestational Age
IUFD	Intra-uterine Fetal Demise
